# Ratio of hemoglobin to red cell distribution width: an inflammatory predictor of survival in AIDS-related DLBCL

**DOI:** 10.3389/fimmu.2024.1354325

**Published:** 2024-02-15

**Authors:** Juanjuan Chen, Yihua Wu, Han Zhao, Guangjing Ruan, Shanfang Qin

**Affiliations:** ^1^ Department of Infectious Diseases, Nanfang Hospital, Southern Medical University, Guangzhou, China; ^2^ State Key Laboratory of Organ Failure Research, Key Laboratory of Infectious Diseases Research in South China, Ministry of Education, Guangdong Provincial Key Laboratory of Viral Hepatitis Research, Guangdong Provincial Clinical Research Center for Viral Hepatitis, Guangdong Institute of Hepatology, Guangzhou, China; ^3^ Infectious Diseases Center, Guangzhou Eighth People’s Hospital, Guangzhou Medical University, Guangzhou, China; ^4^ Guangxi AIDS Clinical Treatment Center, The Fourth People’s Hospital of Nanning, Nanning, China; ^5^ Guangxi AIDS Diagnosis and Treatment Quality Control Center, Chest Hospital of Guangxi Zhuang Autonomous Region, Liuzhou, China

**Keywords:** Hb/RDW ratio, aids, diffuse large B-cell lymphoma, prognostic biomarker, inflammatory index

## Abstract

**Background:**

Despite the introduction of combined antiretroviral therapy, AIDS-related diffuse large B-cell lymphoma (AR-DLBCL) remains a prominent cancer among individuals living with HIV with a suboptimal prognosis. Identifying independent prognostic markers could improve risk stratification.

**Methods:**

In this multicenter retrospective cohort study spanning years 2011 to 2019, 153 eligible patients with AR-DLBCL were examined. Overall survival (OS) factors were analyzed using Kaplan–Meier curves, and univariate and multivariate Cox proportional hazards models. The discriminatory ability of the risk score was evaluated by examining the area under the receiver operating characteristic curve.

**Results:**

The study included 153 patients with a median age of 47 years (interquartile range [IQR] 39–58), 83.7% of whom were men. The median follow-up was 12.0 months (95% confidence interval [CI], 8.5–15.5), with an OS rate of 35.9%. Among the potential inflammatory markers examined, only the ratio of hemoglobin (g/dL) to red cell distribution width (%) (Hb/RDW) emerged as an independent prognostic parameter for OS in the training (hazard ratios [HR] = 2.645, 95% CI = 1.267–5.522, *P* = 0.010) and validation cohorts (HR = 2.645, 95% CI = 1.267–5.522, *P* = 0.010). A lower Hb/RDW ratio was strongly correlated with adverse clinical factors, including advanced Ann Arbor stage, increased extranodal sites, reduced CD4 count, elevated lactate dehydrogenase levels, poorer Eastern Cooperative Oncology Group performance status (ECOG PS), and a higher International Prognostic Index (IPI) score. The addition of the Hb/RDW ratio to the IPI produced a highly discriminatory prognostic composite score, termed Hb/RDW-IPI.

**Conclusion:**

We identified a cost-effective and readily available inflammatory biomarker, the Hb/RDW ratio, as an independent predictor of outcomes in patients with AR-DLBCL. Its integration into the IPI score partially improves prognostic accuracy.

## 1 Introduction

People living with HIV (PLWH) face an increased lifetime risk of specific cancers, notably AIDS-related diffuse large B-cell lymphoma (AR-DLBCL), despite effective HIV management with combined antiretroviral therapy (cART) (1). AR-DLBCL, characterized by intricate phenotypic and genetic features, often presents aggressive clinical manifestations, heightened pathogenic heterogeneity, higher relapse rates, and an unfavorable prognosis compared to the general population (2–5). While remarkable progress has been made in the treatment approaches for HIV and DLBCL in recent decades (6–11), early and rapid prognostic assessment is crucial for developing more effective individualized first-line treatment for patients with AR-DLBCL. In the era of targeted treatment and immunochemotherapy, the prognostic significance of the International Prognostic Index (IPI)-based score has diminished, underscoring the need for reliable, easily accessible, simple, and cost-effective prognostic indicators.

Severe systemic inflammation and immune dysregulation are hallmark features of HIV (12, 13). Recent evidence suggests that, beyond the inherent characteristics of cancer clones, tumor-related inflammation is associated with clinical behavior (14). The red cell distribution width ratio (%, RDW), a component of the complete blood cell count, measures the heterogeneity in the size of circulating red blood cells, offering insights into the severity of inflammation in conditions such as malignancy, cardiovascular diseases, and sepsis (15–20). A novel derived inflammatory biomarker, the ratio of hemoglobin (g/dL) to red cell distribution width (%), closely correlates with the presence of systemic inflammation and significantly enhances mortality prediction in cancer, epidermal necrolysis, and senescence (21, 22). However, it remains to be defined whether evaluating inflammation can provide additional prognostic information, improve prognostic stratification, and assist in treatment decisions for AR-DLBCL.

Therefore, we explored the association between the Hb/RDW ratio and overall survival (OS) and assessed the incremental prognostic value of this new inflammatory marker when combined with the International Prognostic Index (IPI) in a contemporary cohort of patients with AR-DLBCL.

## 2 Methods

### 2.1 Patients

**Figure 1:**
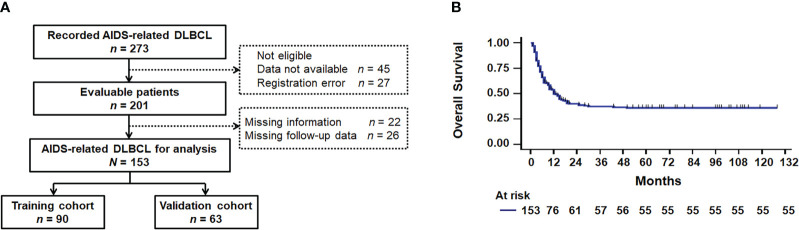
Survival analysis. **(A)** Flowchart diagram. **(B)** Kaplan–Meier survival curve for overall survival based on 153 patients with AIDS-related DLBCL. DLBCL, diffuse large B-cell lymphoma.

This multicenter retrospective study was conducted at Nanfang Hospital Affiliated with Southern Medical University, Guangzhou Eighth People’s Hospital, Fourth Hospital of Nanning, and Chest Hospital of Guangxi Zhuang Autonomous Region in China. We enrolled 273 patients with initial AIDS-related DLBCL from January 2011 to December 2019, based on the 2008 WHO classification (23). The exclusion criteria consisted of patients with transformed DLBCL, primary central nervous system DLBCL, primary cutaneous DLBCL, and patients lacking complete physical examination, detailed disease history, treatment records, results, and follow-up data. A total of 153 patients (90 in the training cohort and 63 in the validation cohort) met the eligibility criteria (**Figure 1A**). The training cohort included patients from Nanfang Hospital and Chest Hospital of Guangxi Zhuang Autonomous Region, the other two hospitals contributed to the external validation cohort. Detailed inclusion and exclusion criteria are provided in the **Supplementary Methods**. The study obtained approval from the Ethics Committees of Nanfang Hospital (NFEC-2021-178), Guangzhou Eighth People’s Hospital (202210222), the Fourth People’s Hospital of Nanning ([2019]39), and the Chest Hospital of Guangxi Zhuang Autonomous Region (2022-022). Written informed consent was obtained from all participants in accordance with the World Medical Association Declaration of Helsinki.

### 2.2 Data collection and study endpoint

Baseline characteristics, including age, sex, lactate dehydrogenase (LDH) levels, Eastern Cooperative Oncology Group performance status (ECOG PS), Ann Arbor stage, extranodal sites, Hb/RDW ratio, presence of B-symptoms, bulky disease, cell of origin, prior history of HIV, cART, CD4 levels, and the CD4/CD8 ratio, were collected. The primary endpoint of this study was OS, defined as the time from diagnosis to death from any cause.

### 2.3 Construction of the Hb/RDW-IPI risk score

The Hb/RDW ratio was integrated into the IPI score, and the incremental prognostic value was evaluated by comparing the area under the curve (AUC) of the receiver operating characteristic (ROC) curve between the IPI and the Hb/RDW-IPI risk score.

### 2.4 Statistical analysis

The optimal cutoff values for the biomarkers were determined using ROC curve analysis and the Youden index. Wilcoxon rank-sum tests or one-way analysis of variance on ranks were employed to compare continuous variables. Pearson’s and nonparametric Spearman’s correlations were used to assess the correlation between continuous variables. The Kaplan–Meier method was used to visualize the time-to-event variables, and log-rank tests were used to compare survival between cohorts. Univariate analysis identified potential prognostic variables correlated with OS. Variables significant at *P* < 0.05 in univariate analyses were included in the multivariable analysis using the Cox proportional hazards model. The association between variables and survival was summarized by the Hazard ratio (HR) and 95% confidence intervals (CI). Linear correlation analysis and the Student’s t-test were used to assess the relationship of the Hb/RDW ratio with other factors. Pearson’s chi-square was used to analyze the distributions of clinical characteristics between different groups. SPSS v.25.0 was used for all statistical analyses. All tests were two-sided, and significance was defined as a *P* < 0.05.

## 3 Results

### 3.1 Patient characteristics

Between January 2011 and December 2019, four centers in southern China registered 273 cases of AIDS-related DLBCL, with 153 confirmed eligible (**Figure 1A**). A comparison between the training cohort (n = 90) and the validation cohort (n = 63) revealed no significant differences in demographics and other parameters (**Table 1**). The median duration of follow-up was 12.0 months (95% CI, 8.5–15.5; range, 1 to 104 months), and the OS rate was 35.9% (**Figure 1B**, **Supplementary Figure 1**). Specifically, patients with AR-DLBCL exhibited 1-, 2-, and 3-year OS rates of 49.7%, 39.9%, and 37.3%, respectively. The median age was 47 years [interquartile range (IQR) 39–58], with 128 patients (83.7%) being male. Among them, 31 individuals (20.3%) were over 60 years old, 118 (77.1%) had LDH > upper limit of normal (ULN), 76 (49.7%) had ECOG PS > 1, 78 (51.0%) had Ann Arbor stage III/IV disease, 44 (28.8%) had extranodal disease at > 1 site (**Table 1**), and 62 (40.6%) had IPI high-intermediate–risk and high–risk scores (**Supplementary Table 1**). The median Hb/RDW ratio was 8.4 (IQR 6.0–10.1). The optimal cutoff for the Hb/RDW ratio, determined through ROC curve analysis (**Supplementary Figure 3A**), was 8.5 (AUC = 0.737, 95% CI = 0.658–0.816, *P* < 0.001). Among the patients, 80 (52.3%) had a Hb/RDW ratio < 8.5. B symptoms were found in 25 patients (16.3%), bulky disease (> 7.5 cm) was documented in 57 individuals (37.3%), and the germinal center B-cell (GCB) subtype was observed in 81 patients (52.9%). Before the onset of AR-DLBCL, 38 out of 153 patients (24.8%) had a history of HIV infection. Among them, 29 (76.3%) were on cART for over 3 months, and 5 (13.2%) lacked treatment records. Of those on cART, 9 out of 29 (31.0%) had an undetectable HIV-1 viral load, while 9 (31.0%) had unknown viral load levels, partly due to incomplete historical records. Additionally, 97 patients (63.4%) had received cART at the baseline of lymphoma treatment. The median CD4+ T cell count and the CD4/CD8 ratio were 171 cells/µL (IQR 73–274) and 0.26 (IQR 0.15–0.47), respectively (**Table 1**).

**Table 1:** AIDS-related DLBCL patient characteristics.

Characteristic, No. (%)	Total (N = 153)	Training (*n* = 90)	Validation (*n* = 63)	*P* value
Male sex	128 (83.7)	74 (82.2)	54 (85.7)	0.565
Age, years (median, IQR)	47 [39–58]	47 [39–56]	47 [37–60]	0.878
> 60	31 (20.3)	15 (16.7)	16 (25.4)	0.186
LDH, U/L (median, IQR)	449 [255–846]	473 [255–1106]	382 [254–789]	0.263
> ULN	118 (77.1)	69 (76.7)	49 (77.8)	0.872
ECOG PS > 1	76 (49.7)	46 (51.1)	30 (47.6)	0.671
Ann Arbor stage III/IV	78 (51.0)	45 (50.0)	33 (52.4)	0.772
Extranodal sites > 1	44 (28.8)	23 (25.6)	21 (33.3)	0.296
Hb/RDW ratio (median, IQR)	8.4 [6.0–10.1]	8.8 [6.1–10.2]	8.0 [6.0–10.0]	0.410
< 8.5	80 (52.3)	43 (47.8)	37 (58.7)	0.182
B symptoms (Yes)	25 (16.3)	16 (17.8)	9 (14.3)	0.565
Bulky disease (Yes)	57 (37.3)	35 (38.9)	22 (34.9)	0.617
Cell of origin				0.213
GCB	81 (52.9)	53 (58.9)	28 (44.4)	
Inconclusive	49 (32.0)	25 (27.8)	24 (38.1)	
Prior history of HIV (Yes)	38 (24.8)	25 (27.8)	13 (20.6)	0.314
cART at baseline				0.599
Yes	97 (63.4)	60 (66.7)	37 (58.7)	
Inconclusive	36 (23.5)	19 (21.1)	17 (27.0)	
CD4 count, cells/μL (median, IQR)	171 [73–274]	167 [84–271]	174 [64–305]	0.984
< 200	87 (56.9)	52 (57.8)	35 (55.6)	0.785
CD4/CD8 (median, IQR)	0.26 [0.15–0.47]	0.29 [0.17–0.48]	0.25 [0.14–0.41]	0.490
< 0.41	108 (70.6)	62 (68.9)	46 (73.0)	0.581

DLBCL, diffuse large B-cell lymphoma; IQR, interquartile range; LDH, lactate dehydrogenase; ULN, upper limit of normal; ECOG PS, Eastern Cooperative Oncology Group performance status; Hb/RDW ratio, ratio of hemoglobin (g/dL) to red cell distribution width (%); GCB, germinal center B-cell; cART, combined antiretroviral therapy.

### 3.2 The Hb/RDW ratio was an independent prognostic marker for OS

In the training cohort, univariate analysis revealed that age, LDH, Hb/RDW ratio, CD4 count, and the CD4/CD8 ratio were predictive of OS (**Table 2**). In multivariable analysis, considering DLBCL, HIV, and inflammation-related factors, age (HR = 2.873, 95% CI = 1.522–5.421, *P* = 0.001), LDH levels (HR = 2.421, 95% CI = 1.058–5.537, *P* = 0.036), the Hb/RDW ratio (HR = 2.645, 95% CI = 1.267–5.522, *P* = 0.010), and the CD4/CD8 ratio (HR = 2.151, 95% CI = 1.076–4.301, *P* = 0.030) remained independent predictors of clinical outcome (**Table 2**, **Supplementary Figure 2**). In particular, only the Hb/RDW ratio (HR = 2.645, 95% CI = 1.267–5.522, *P* = 0.010) retained its independent prognostic value in the external validation cohort (**Table 2**).

**Table 2:** Cox regressions for overall survival in AIDS-related DLBCL.

Characteristic	Training (*n* = 90)				Validation (*n* = 63)			
	Univariate		Multivariate		Univariate		Multivariate	
	HR (95% CI)	*P* value	HR (95% CI)	*P* value	HR (95% CI)	*P* value	HR (95% CI)	*P* value
Male sex	1.122 (0.550–2.290)	0.752			1.271 (0.499–3.237)	0.615		
Age > 60 years	2.961 (1.621–5.409)	**0.001**	2.873 (1.522–5.421)	**0.001**	1.940 (1.005–3.745)	**0.048**	1.614 (0.801–3.254)	0.181
LDH > ULN	3.052 (1.378–6.758)	**0.006**	2.421 (1.058–5.537)	**0.036**	1.810 (0.835–3.924)	0.133	1.395 (0.617–3.155)	0.424
ECOG PS > 1	1.414 (0.832–2.402)	0.200	1.502 (0.870–2.594)	0.144	1.598 (0.868–2.940)	0.132	1.303 (0.641–2.652)	0.465
Ann Arbor stage III/IV	1.072 (0.634–1.812)	0.795	1.196 (0.608–2.352)	0.604	1.618 (0.871–3.005)	0.128	1.418 (0.620–3.243)	0.408
Extranodal sites > 1	0.992 (0.549–1.795)	0.980	0.781 (0.366–1.666)	0.522	1.250 (0.664–2.354)	0.489	0.664 (0.277–1.593)	0.359
Hb/RDW ratio < 8.5	1.936 (1.134–3.304)	**0.016**	1.897 (1.094–3.290)	**0.023**	3.219 (1.596–6.494)	**0.001**	2.645 (1.267–5.522)	**0.010**
B symptoms (Yes)	1.671 (0.880–3.173)	0.117			1.652 (0.731–3.730)	0.227		
Bulky disease (Yes)	1.538 (0.907–2.608)	0.110			3.197 (1.725–5.926)	**0.001**		
Prior history of HIV (Yes)	0.902 (0.505–1.613)	0.728			1.340 (0.658–2.728)	0.420		
cART at baseline (Yes)	1.364 (0.630–2.956)	0.431			0.719 (0.292–1.773)	0.474		
CD4 count < 200, cells/μL	1.896 (1.078–3.328)	**0.026**			1.468 (0.779–2.768)	0.235		
CD4/CD8 < 0.41	2.158 (1.137–4.097)	**0.019**	2.151 (1.076–4.301)	**0.030**	1.729 (0.799–3.742)	0.165	1.543 (0.678–3.511)	0.302

DLBCL, diffuse large B-cell lymphoma; HR, hazard ratio; CI, confidence interval; LDH, lactate dehydrogenase; ULN, upper limit of normal; ECOG PS, Eastern Cooperative Oncology Group performance status; Hb/RDW ratio, ratio of hemoglobin (g/dL) to red cell distribution width (%); cART, combined antiretroviral therapy.

The bold means P < 0.05.

### 3.3 The Hb/RDW ratio as a novel inflammatory factor for prognosis

**Figure 2:**
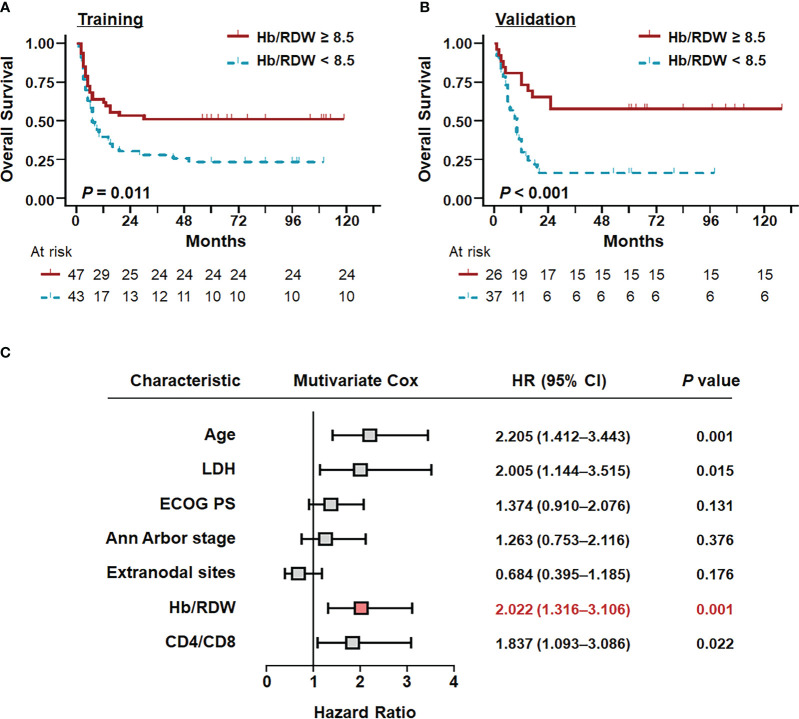
Survival curves stratified by the independent predictors in patients with AIDS-related DLBCL. Overall survival of patients with AR-DLBCL stratified by the Hb/RDW ratio in the **(A)** training and **(B)** validation cohorts, as determined by Kaplan–Meier survival analyses. **(C)** Prognostic factors associated with overall survival in patients with AIDS-related DLBCL in the total cohort. Hb/RDW, ratio of hemoglobin (g/dL) to red cell distribution width (%); LDH, lactate dehydrogenase; ECOG PS, Eastern Cooperative Oncology Group performance status.

**Figure 3:**
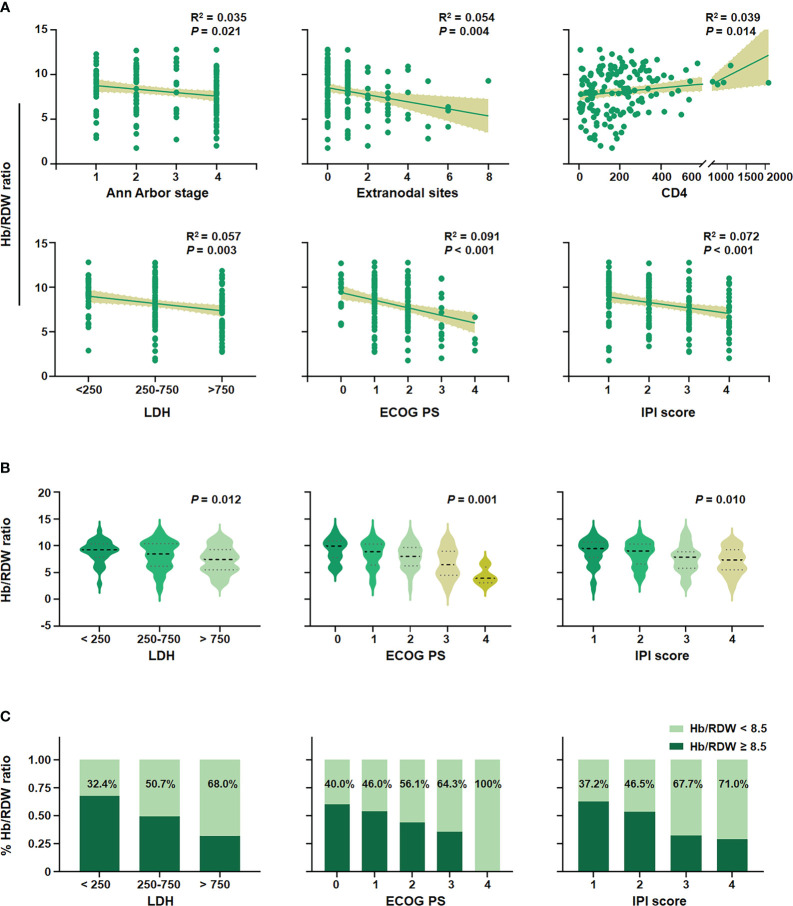
Correlation of the Hb/RDW ratio with clinical variables in AIDS-related DLBCL. **(A)** Association between the level of the Hb/RDW ratio and the Ann Arbor stage, extranodal sites, CD4, LDH, ECOG PS, and IPI score. **(B)** The average level of the Hb/RDW ratio in the LDH groups, the ECOG PS groups, and IPI score groups. **(C)** Proportions of Hb/RDW ratio in LDH groups, ECOG PS groups, and IPI score groups. Hb/RDW, ratio of hemoglobin (g/dL) to red cell distribution width (%); LDH, lactate dehydrogenase; ECOG PS, Eastern Cooperative Oncology Group performance status; IPI, International Prognostic Index.

Kaplan–Meier analysis highlighted a significant association between a low Hb/RDW ratio and poor outcomes (**Figures 2A, B**). Similarly, this novel inflammatory marker, the Hb/RDW ratio (HR = 2.022, 95% CI = 1.316–3.106, *P* = 0.001), consistently demonstrated prognostic significance in the overall cohort (**Figure 2C**). An lower Hb/RDW ratio was associated with adverse clinical factors (**Figure 3A**), including the advanced Ann Arbor stage (R^2 = ^0.035, *P* = 0.021), increased extranodal sites (R^2 = ^0.054, *P* = 0.004), reduced CD4 count (R^2 = ^0.039, *P* = 0.014), elevated levels of LDH (*P* = 0.012), poorer ECOG PS (*P* = 0.001), and higher IPI score (*P* = 0.010). The chi-square test (**Table 3**) demonstrated a significant correlation between the Hb/RDW ratio and LDH levels (*P* = 0.015), Ann Arbor stage (*P* = 0.044), and the extranodal sites (*P* = 0.012).

**Table 3:** Comparison of AIDS-related DLBCL patient characteristics between the Hb/RDW stratification.

Characteristic	Hb/RDW ratio		
	≥ 8.5 (n = 73, %)	< 8.5 (n = 80, %)	*P* value
Male	62 (84.9)	66 (82.5)	0.685
Age > 60 years	12 (16.4)	19 (23.8)	0.261
LDH > ULN	50 (68.5)	68 (85.0)	**0.015**
ECOG PS > 1	31 (42.5)	45 (56.3)	0.089
Ann Arbor stage III/IV	31 (42.5)	47 (58.8)	**0.044**
Extranodal sites > 1	14 (19.2)	30 (37.5)	**0.012**
B symptoms (Yes)	9 (12.3)	16 (20.0)	0.200
Bulky disease (Yes)	26 (35.6)	31 (38.8)	0.689
COO (GCB)	43 (58.9)	38 (47.5)	0.173
Prior history of HIV (Yes)	21 (28.8)	17 (21.3)	0.282
cART at baseline (Yes)	49 (67.1)	48 (60.0)	0.482
CD4 count < 200, cells/μL	36 (49.3)	51 (63.8)	0.072
CD4/CD8 < 0.41	51 (69.9)	57 (71.3)	0.851

DLBCL, diffuse large B-cell lymphoma; Hb/RDW ratio, ratio of hemoglobin (g/dL) to red cell distribution width (%); LDH, lactate dehydrogenase; ULN, upper limit of normal; ECOG PS, Eastern Cooperative Oncology Group performance status; COO, cell of origin; GCB, germinal center B-cell; cART, combined antiretroviral therapy.

The bold means P < 0.05.

Furthermore, we conducted additional analyses to evaluate the significance of the Hb/RDW ratio within different subgroups based on the LDH, ECOG PS, and IPI scores (**Figures 3B, C**). Among the AR-DLBCL population, 32.4%, 50.7%, and 68.0% exhibited a low Hb/RDW ratio in groups with LDH levels < 250, 250-750, and > 750 U/L, respectively (*P* = 0.012). Similarly, among patients with ECOG PS 0, 1, 2, 3, and 4, the proportion of patients with a reduced Hb/RDW ratio were 40.0%, 46.0%, 56.1%, 64.3%, and 100%, respectively (*P* = 0.001). A comparable trend was observed in the IPI score groups, with the percentages of individuals having a low Hb/RDW ratio at 37.2%, 46.5%, 67.7%, and 71.0% in the IPI score 1, 2, 3, and 4 groups, respectively (*P* = 0.010).

### 3.4 Incorporation of the Hb/RDW ratio into the IPI for OS prediction

**Figure 4:**
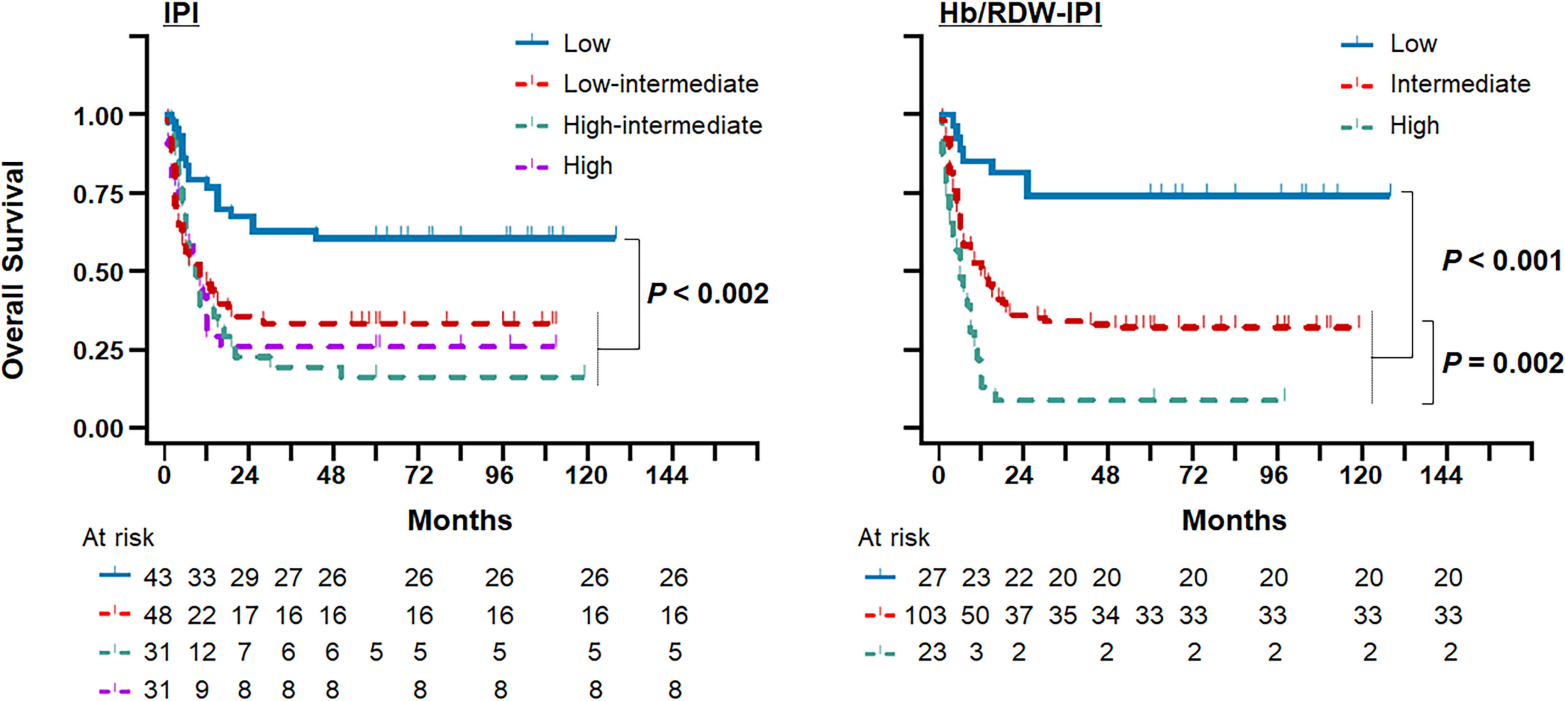
Incorporation of the Hb/RDW ratio into the IPI for the prediction of overall survival in the AIDS-related DLBCL setting. Kaplan–Meier plots comparing the Hb/RDW-IPI score with the IPI score in the total cohort. Hb/RDW, ratio of hemoglobin (g/dL) to red cell distribution width (%); IPI, International Prognostic Index.

Incorporating the Hb/RDW ratio into the IPI score defined the Hb/RDW-IPI scores, with each variable scored as 1 based on the hazard ratio of each category in the total cohort (**Figure 2C**, 16), which allowed us to significantly improve survival prediction (**Figure 4**). The entire cohort was accurately classified into three risk groups: low-risk (scores: 0–1; 17.6%), intermediate-risk (scores: 2–4; 67.3%), and high-risk (scores: 5–6; 15.0%), corresponding to OS rates of 74.1%, 32.0%, and 8.7%, respectively. The combined Hb/RDW-IPI demonstrated a statistically significant stratification of the three risk groups (*P* < 0.001) and exhibited superior distinction for the intermediate- and high-risk groups compared to the IPI score alone. Hb/RDW-IPI showed a moderate improvement in discrimination compared to the original IPI alone (**Supplementary Figure 3B**, AUC [95% CI]: Hb/RDW-IPI, 0.70 [0.61–0.78] vs. IPI, 0.67 [0.58–0.77]).

## 4 Discussion

AIDS-related DLBCL is recognized for its aggressive nature and distinctive clinical characteristics (24). In this study, we established that the Hb/RDW ratio serves as an independent prognostic indicator for AR-DLBCL, significantly enhancing prognostic discrimination when incorporated with the IPI score for risk stratification.

HIV-positive DLBCL typically manifests with aggressive features, characterized by poor performance status, elevated LDH levels, B symptoms, advanced-stage, high IPI scores, and a high incidence of extranodal and central nervous system (CNS) involvement (25–27). In our analyses, we observed that 49.7% of patients had an ECOG PS > 1, 77.1% had elevated LDH, 16.3% exhibited B symptoms, 28.8% had extranodal sites > 1, 51.0% were classified as advanced-stage (stage III/IV), and 40.6% were in the IPI ≥ 3 group, aligning with findings in previous studies (24, 27, 28).

At Nanfang Hospital, one of the four hospitals in our cohort, the OS rate for AR-DLBCL patients was 75.9% (data not shown), while the OS rate for the total cohort was 35.9%. This discrepancy can be attributed to the retrospective, non-randomized, real-world comparison across diverse regions with varying access to medical resources. Furthermore, chemoimmunotherapy may not be accessible in some cases (29). Hence, it is essential to consider real-life conditions in less developed countries and regions when investigating new biomarkers.

Chronic inflammation and infections have been identified as etiological factors of DLBCL (30, 31). Previous studies have shown a positive correlation between the Hb/RDW ratio or the RDW ratio and inflammatory markers, such as C-reactive protein, tumor necrosis factor alpha, and the erythrocyte sedimentation rate, indicating that the Hb/RDW ratio may reflect an underlying inflammatory response (22, 32–34). However, there is currently no literature reporting on the prognostic significance of the Hb/RDW ratio in patients with AR-DLBCL. In this multicenter retrospective study, we validated the beneficial role of the Hb/RDW ratio in evaluating the prognosis of patients with AR-DLBCL. Our findings indicated that patients with a low Hb/RDW ratio were frequently associated with advanced Ann Arbor stage, increased extranodal sites, lower CD4 count, higher LDH levels, poorer ECOG PS, and a higher IPI score. Additionally, the Hb/RDW ratio is a readily available biomarker that can be calculated from values obtained from the complete blood cell count, a test routinely performed in clinical practice. Thus the Hb/RDW ratio does not require expensive instruments, complex calculations, or additional costs.

RDW, an indicator of tumor-associated inflammatory responses, has been proven to be associated with the prognosis of certain cancers (35). However, the specific mechanisms underlying the association between the Hb/RDW ratio and AR-DLBCL mortality remain unclear. There is increasing evidence that inflammation is a systemic factor that disrupts erythrocyte homeostasis (17, 22). Short telomere length, inflammation, oxidative stress, poor nutritional status, erythrocyte fragmentation, dyslipidemia, hypertension, and abnormality in erythropoietin function may represent potential biological mechanisms (32). Patients with AR-DLBCL are well-known to experience chronic inflammation, a high tumor burden, and malnutrition, which may lead to profound deregulation of erythrocyte homeostasis, including impaired erythropoiesis, abnormal erythrocyte metabolism, and survival (35). These speculations require further verification through additional fundamental research.

This study is limited by its small sample size. Prospective investigations with larger samples and more robust statistical analyses are necessary to draw definitive conclusions regarding the utility of the Hb/RDW ratio as a marker for AR-DLBCL. In addition, the inclusion of patients diagnosed and treated in different institutions introduces inherent biases in this retrospective study design. Our real-world study included low- and middle-income populations from underdeveloped regions, lacking treatment resources, trained specialists, supportive care, and the affordability of rituximab. Furthermore, the study was conducted in a Chinese population, and warrants further research to validate the utility of the Hb/RDW ratio in other populations and settings.

In conclusion, the Hb/RDW ratio, an inexpensive and easily available inflammatory biomarker, emerges as a novel prognostic parameter in patients with AR-DLBCL. Integrating the Hb/RDW ratio into the IPI improves the mortality prognostication for AR-DLBCL to some extent. Accurate risk stratification is crucial to improve patient assessment and management and will provide more precise guidance on individualized treatment.

## Data availability statement

The original contributions presented in the study are included in the article/**Supplementary Material**. Further inquiries can be directed to the corresponding author.

## Ethics statement

The study received approval from the Ethics Committees of Nanfang Hospital (NFEC-2021-178), Guangzhou Eighth People’s Hospital (202210222), Fourth People’s Hospital of Nanning ([2019]39), and Chest Hospital of Guangxi Zhuang Autonomous Region (2022-022). The studies were conducted in accordance with the local legislation and institutional requirements. The participants provided their written informed consent to participate in this study.

## Author contributions

JC: Conceptualization, Funding acquisition, Methodology, Project administration, Resources, Supervision, Writing – original draft, Writing – review & editing. YW: Data curation, Formal analysis, Investigation, Methodology, Validation, Writing – original draft. HZ: Data curation, Writing – original draft. GR: Data curation, Writing – original draft. SQ: Data curation, Writing – original draft.
