# Supplementary material for: Ratio of hemoglobin to red cell distribution width: an inflammatory predictor of survival in AIDS-related DLBCL
Source: Front Immunol. 2024 Feb 15;15:1354325. doi: 10.3389/fimmu.2024.1354325 (PMC10901994; doi:10.3389/fimmu.2024.1354325)
Supplement: Supplementary file 1 [file DataSheet_1.pdf]

Supplementary Material

1 Supplementary methods

Inclusion Criteria:

1. HIV-positive

2. Age ≥ 18 years

3. Previously untreated

4. Histologically confirmed CD20 positive diffuse large B-cell lymphoma between January 2011 and December 2019

5. Negative pregnancy

Exclusion Criteria:

1. Transformed DLBCL

2. Primary central nervous system DLBCL

3. Primary cutaneous DLBCL

4. Lack of a complete physical examination, no detailed history of disease, treatments, outcomes, and follow-up data.

2 Supplementary Figures and Tables

2.1 Supplementary Figures

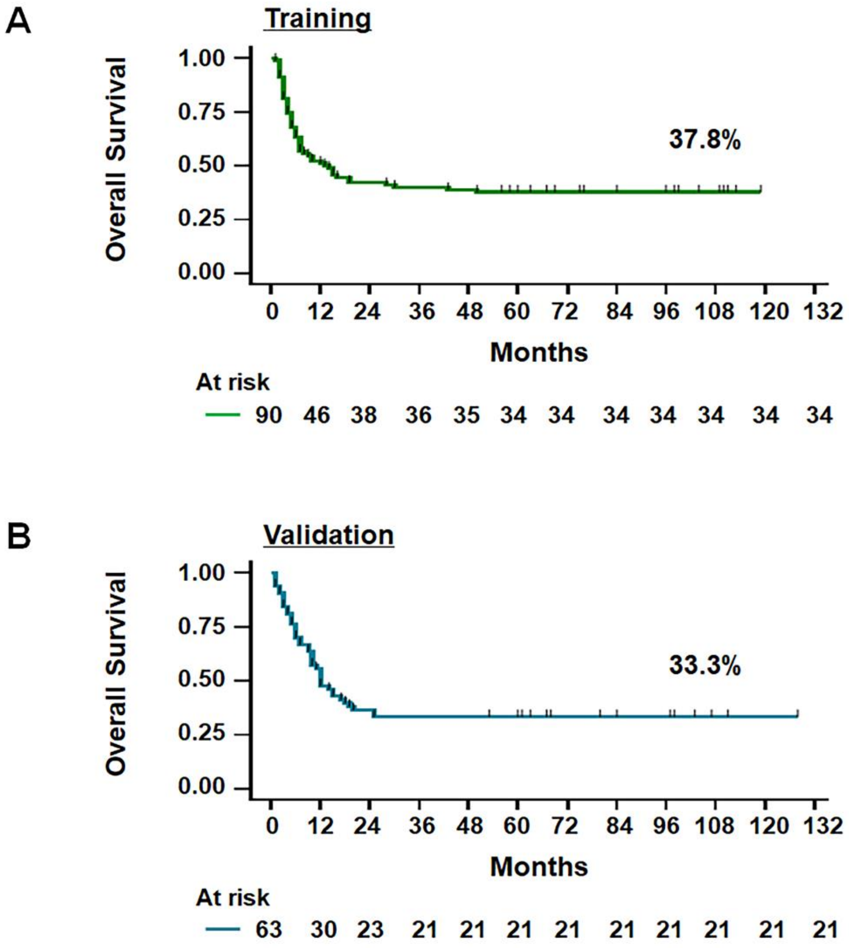

Supplementary Figure 1. Survival analysis. Kaplan–Meier survival curves for the overall survival of patients with AIDS-related DLBCL in the **(A)** training and **(B)** validation cohorts. Abbreviations: DLBCL, diffuse large B-cell lymphoma.

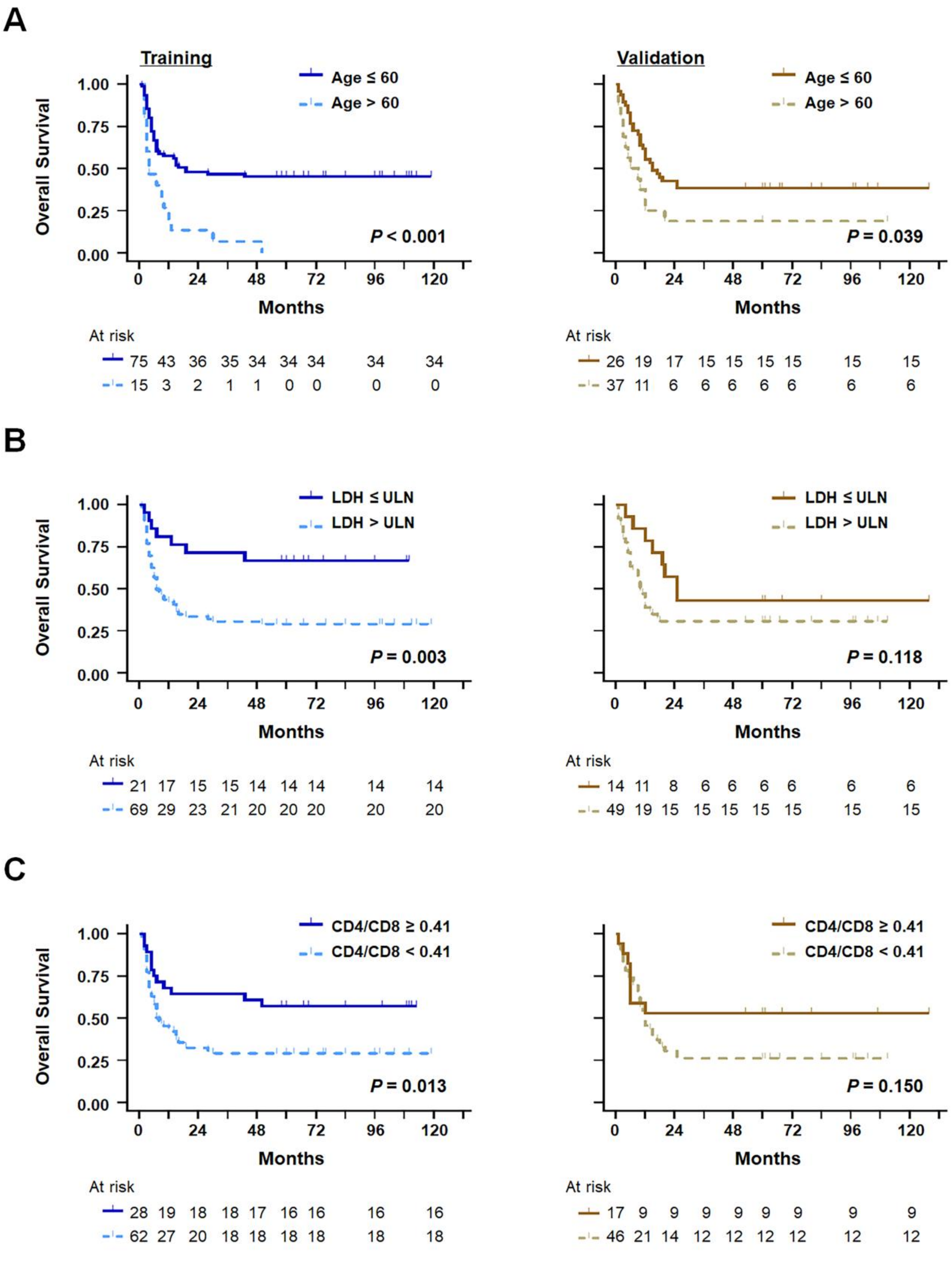

Supplementary Figure 2. Survival curves stratified by the independent predictor in patients with AIDS-related DLBCL. Overall survival of AR-DLBCL patients stratified by **(A)** age, **(B)** lactate dehydrogenase (LDH), and **(C)** CD4/CD8 ratio in the training (left) and validation (right) cohorts, as determined by Kaplan–Meier survival analyses. Abbreviations: LDH, lactate dehydrogenase.

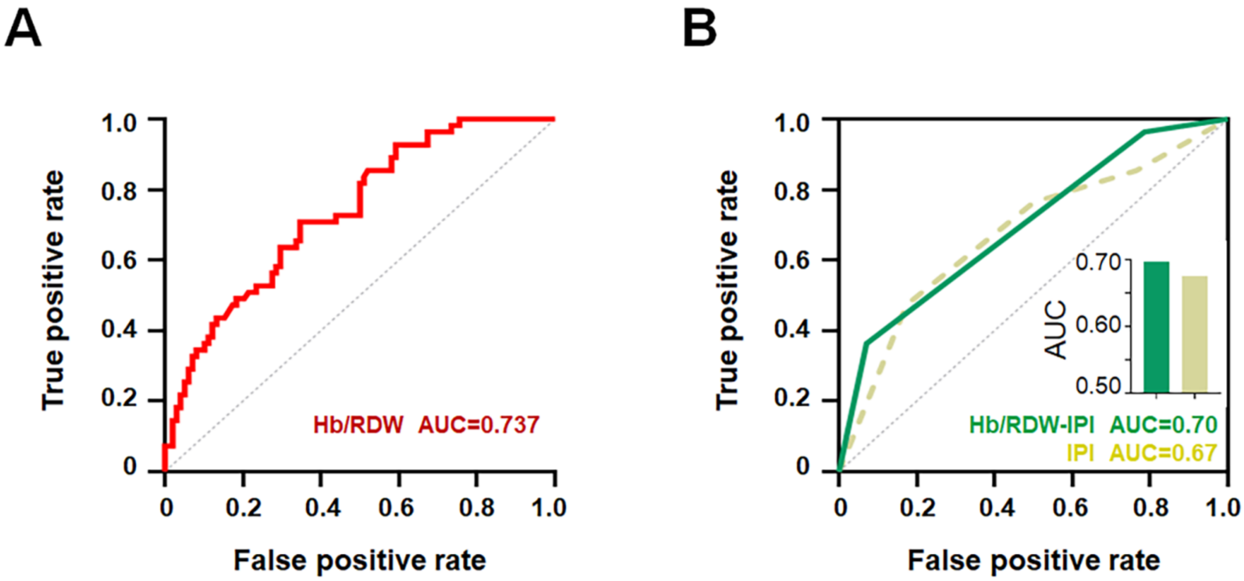

Supplementary Figure 3. Comparison of the predictive performance. **(A)** The receiver operating characteristic (ROC) curve and the area under curve (AUC) of the Hb/RDW ratio. **(B)** The ROC curves and AUC were used to assess the predictive performance of the Hb/RDW-IPI score compared with the IPI score in the total cohort. Abbreviations: Hb/RDW, the ratio of hemoglobin to red cell distribution width; IPI, International Prognostic Index.

2.2 Supplementary Tables

Supplementary Table 1. The IPI score, Hb/RDW-IPI score, first-line combination anti-retroviral therapy, anti-tumor therapy and response of AIDS-related DLBCL patients

**Characteristic, No. (%)**	**Total (N = 153)**	**Training (*n* = 90)**	**Validation (*n* = 63)**	***P* value**
cART				0.427
NRTIs + NNRTIs	70 (45.8)	41 (45.6)	29 (46.0)	
NRTIs + PIs	13 (8.5)	8 (8.9)	5 (7.9)	
NRTIs + INSTIs	14 (9.2)	11 (12.2)	3 (4.8)	
Untreated	20 (13.1)	11 (12.2)	9 (14.3)	
Inconclusive	36 (23.5)	19 (21.1)	17 (27.0)	
IPI				0.003
Low–risk (0-1)	43 (28.1)	23 (25.6)	20 (31.7)	
Low-intermediate–risk (2)	48 (31.4)	32 (35.6)	16 (25.4)	
High-intermediate–risk (3)	31 (20.3)	23 (25.6)	8 (12.7)	
High–risk (4-5)	31 (20.3)	12 (13.3)	19 (30.2)	
Hb/RDW-IPI				0.005
Low risk (0-1)	27 (17.6)	14 (15.6)	13 (20.6)	
Intermediate risk (2-4)	103 (67.3)	68 (75.6)	35 (55.6)	
High risk (5-6)	23 (15.0)	8 (8.9)	15 (23.8)	
Treatment received				0.019
Immunochemotherapy-based	27 (17.6)	18 (20.0)	9 (14.3)	
Chemotherapy-based	113 (73.9)	61 (67.8)	52 (82.5)	
Untreated	13 (8.5)	11 (12.2)	2 (3.2)	
Treatment outcomes				0.039
CR	57 (37.3)	36 (40.0)	21 (33.3)	
PR	25 (16.3)	14 (15.6)	11 (17.5)	
SD	36 (23.5)	16 (17.8)	20 (31.7)	
PD	22 (14.4)	13 (14.4)	9 (14.3)	
Untreated	13 (8.5)	11 (12.2)	2 (3.2)	

Abbreviations: DLBCL, diffuse large B-cell lymphoma; cART, combination antiretroviral therapy; NRTIs, nucleoside/nucleotide reverse transcriptase inhibitors; NNRTIs, non-nucleoside reverse transcriptase inhibitors; PIs, protease inhibitors; INSTIs, integrase strand-transfer inhibitors; IPI, International Prognosis Index; Hb/RDW, the ratio of hemoglobin to red cell distribution width; CR, complete response; PR, partial response; SD, stable disease; PD, progressive disease.
